# Green Microalgae *Scenedesmus Obliquus* Utilization for the Adsorptive Removal of Nonsteroidal Anti-Inflammatory Drugs (NSAIDs) from Water Samples

**DOI:** 10.3390/ijerph17103707

**Published:** 2020-05-25

**Authors:** Andreia Silva, Ricardo N. Coimbra, Carla Escapa, Sónia A. Figueiredo, Olga M. Freitas, Marta Otero

**Affiliations:** 1 REQUIMTE/LAQV, Instituto Superior de Engenharia Do Porto, Politécnico Do Porto, Rua Dr. António Bernardino de Almeida 431, 4200-072 Porto, Portugal; andreia.silva@graq.isep.ipp.pt (A.S.); saf@isep.ipp.pt (S.A.F.); omf@isep.ipp.pt (O.M.F.); 2 Department of Environment and Planning, University of Aveiro, Campus Universitário de Santiago, 3810-193 Aveiro, Portugal; ricardo.coimbra@ua.pt; 3 Department of Applied Chemistry and Physics, Institute of Environment, Natural Resources and Biodiversity (IMARENABIO), Universidad de León, 24071 León, Spain; carla.escapa@unileon.es; 4 Centre for Environmental and Marine Studies (CESAM), University of Aveiro, Campus Universitário de Santiago, 3810-193 Aveiro, Portugal

**Keywords:** nonsteroidal anti-inflammatory drugs, green microalgae, wastewater treatment, adsorption, biorefinery

## Abstract

In view of the valorisation of the green microalga *Scenedesmus obliquus* biomass, it was used for the biosorption of two nonsteroidal anti-inflammatory drugs, namely salicylic acid and ibuprofen, from water. Microalgae biomass was characterized, namely by the determination of the point of zero charge (pH_PZC_), by Fourier transform infrared (FT-IR) analysis, simultaneous thermal analysis (STA) and scanning electron microscopy with energy dispersive spectroscopy (SEM/EDS). Kinetic and equilibrium batch experiments were carried out and results were found to fit the pseudo-second order equation and the Langmuir isotherm model, respectively. The Langmuir maximum capacity determined for salicylic acid (63 mg g^−1^) was larger than for ibuprofen (12 mg g^−1^), which was also verified for a commercial activated carbon used as reference (with capacities of 250 and 147 mg g^−1^, respectively). For both pharmaceuticals, the determination of thermodynamic parameters allowed us to infer that adsorption onto microalgae biomass was spontaneous, favourable and exothermic. Furthermore, based on the biomass characterization after adsorption and energy associated with the process, it was deduced that the removal of salicylic acid and ibuprofen by *Scenedesmus obliquus* biomass occurred by physical interaction.

## 1. Introduction

Nonsteroidal anti-inflammatory drugs (NSAIDs) constitute a class of pharmaceuticals able to suppress the production of prostaglandins by inhibiting cyclooxygenase (COX), an enzyme required for prostaglandin biosynthesis. Their main therapeutic actions are anti-inflammatory (inflammation-alleviating), analgesic (pain-killing) and antipyretic (fever-reducing) [1]. They are among the most widely used medications in the world because of their demonstrated efficacy and because they can be acquired over-the-counter, that is, without medical prescription from health specialists [1,2]. Therefore, tons of these pharmaceuticals are daily consumed in the world, this consumption having a rising trend due to the population growth and increasing life expectancy [3]. After NSAIDs consumption, pharmaceutically active compounds are released from the body through urine and faeces, either in their original form or as metabolites [4] and make way to wastewater treatment plants (WWTPs) [5]. Furthermore, the bad practice of flushing unused or expired medicines down the toilet also contributes to the presence of NSAIDs in sewage [6]. In WWTPs the sewage is subjected to successive treatments, which aim is the fulfilment of regulations regarding the discharge of treated water in the aquatic environment. However, since pharmaceuticals are not regulated, treatments are not designed for their removal and, consequently, they are still present in effluents from WWTPs [7,8]. In the specific case of NSAIDs, Mlunguza et al. [9] recently revised the literature and indicated that the removal efficiency in WWTPs varies from −174 to 99%, with negative values being related to influent-effluent mismatching, creation of conjugated compounds or release from faeces during water treatment, day-to-day instability, analytic uncertainty and/or desorption from suspended particulate matter. Among NSAIDs, salicylic acid, which is the active form and metabolite of acetylsalicylic acid [10], and ibuprofen are amongst the most consumed and, therefore, most frequently detected and/or at the largest concentrations in wastewater. For example, salicylic acid was recently found to be present in all the water samples (hospital effluent, wastewater treatment plant influent and effluent, and seawater) in several countries [8,11,12,13,14,15,16]. Ibuprofen was also detected in the same type of water samples and in a river grab sample [12,13,14,15,17] and has been highlighted to be the most consumed medicine all over the world [18,19]. Therefore, there is general consensus about the fact that WWTPs are potential sources of pharmaceuticals, including these NSAIDs, for the aquatic environment [7,8,9]. 

General concern exists on the environmental occurrence of pharmaceuticals since these compounds were designed to induce a physiological response [7,8]. Hence, their presence, even at relative low concentrations, may affect nontarget individuals and species. Indeed, pharmaceuticals are among the so-called emerging contaminants (ECs), which are usually defined as compounds that are not currently covered by existing water regulations but are thought to be a threat to environmental ecosystems and human health [20]. For this reason, the need to develop new, effective and sustainable treatments for the removal of pharmaceuticals from water is evident [21]. Moreover, it is expected that legislation on the discharge of pharmaceuticals will come out in the near future [22] and it will be indispensable being able to accomplish regulations, which reinforces the need to investigate about novel, effective, economic and sustainable treatments.

Among the novel approaches for the removal of pharmaceuticals from water, microalgae-based treatments have received growing attention in the last years [23,24,25,26], with promising results in the specific case of NSAIDs removal [27,28,29]. Biodegradation, biosorption and bioaccumulation have been established as the main mechanisms involved in the removal of pharmaceuticals from water by microalgae culturing [26,27,28,29,30]. Indeed, most of the published works in this field are on the uptake of pharmaceuticals by living microalgae [23,24,25,30]. Meanwhile, the employment of non-living microalgae biomass for the adsorptive removal of these pollutants has been barely studied [21,31], with just a few authors addressing the adsorption of NSAIDs [31,32]. This contrasts with the case of other contaminants, such as metals [33,34] or dyes [35,36], for which the good adsorptive performance of microalgae biomass is renowned. In water treatment, the use of dead microalgae biomass as adsorbent has several advantages over living organisms such as: (1) possible storage at room temperature; (2) long durability without losing sorptive properties; (3) absence of toxicity effects that may affect the treatment performance; (4) possibility of desorbing the pollutant and reutilization of microalgal biomass; and (5) lower operational costs, including the need for growth media [26,33,37]. Moreover, due to present knowledge, the adsorptive removal onto dead microalgae biomass has the advantage of not generating transformation products and metabolites with unknown toxicity effects, while in the case of living microalgae it may occur during biodegradation [37,38,39]. 

From the environmental point of view, the utilization of microalgae biomass as alternative green adsorbent to remove pharmaceuticals from water is a biorefinery approach in line with the circular economy principles [37,40,41]. According to these principles, most or all of microalgae potential should be profitably exploited while waste production should be minimized [37]. Furthermore, apart from the low cost and large availability of microalgal biomass, microalgae utilization as adsorbent may be compatible with a previous extraction of the lipid content and also with a posterior thermochemical conversion [42,43], which may increase the prospective of such an application. In this context, the aim of this work was the characterization of *Scenedesmus obliquus* biomass and the study of its utilization as alternative green adsorbent for the removal of two of the most used NSAIDs, namely, salicylic acid and ibuprofen, from water. Biomass of *Scenedesmus obliquus* has already been shown to display high adsorption capacity as compared with other strains [31] and, furthermore, this strain is among the most used for wastewater treatment, possesses high growth rates, is capable of growing under a wide range of conditions [44], which motivated its choice for this study.

## 2. Materials and Methods 

### 2.1. Microalgae Biomass

Living culture material of microalgae Scenedesmus obliquus (SAG 276-1) was purchased from the Sammlung von Algenkulturen der Universität Göttingen (Culture Collection of Algae at Göttingen University, Göttingen, Germany), international acronym SAG. An inoculum of this strain was maintained in Erlenmeyer flasks (250 mL) containing the standard medium Mann and Myers, which is composed of (per litre of distilled water): 1.2 g MgSO_4_·7H_2_O, 1.0 g NaNO_3_, 0.3 CaCl_2_, 0.1 g K_2_HPO_4_, 3.0 × 10^−2^ g Na_2_EDTA, 6.0 × 10^−3^ g H_3_BO_3_, 2.0 × 10^−3^ g FeSO_4_·7H_2_O, 1.4 × 10^−3^ g MnCl_2_, 3.3 × 10^−4^ g ZnSO_4_·7H_2_O, 7.0 × 10^−6^ g Co(NO_3_)_2_·6H_2_O, 2.0 × 10^−6^ g CuSO_4_·5H_2_O. In order to favour microalgae growing, flasks were kept inside a vegetal culture chamber under mild controlled conditions: temperature (25 ± 1 °C), irradiance (175 µE m^−2^ s^−1^), photoperiod (12 h:12 h) and shaking (250 rpm) until reaching a biomass concentration of 0.1 g L^−1^. Then, the culture was transferred to a photobioreactor (PBR) with a volume of 10 L, which was kept in a vegetal culture chamber under controlled optimized conditions for microalgae exponential growth, namely at 27–30 °C, 16 h:8 h photoperiod and 650 µE m^−2^ s^−1^ irradiance. The PBR was aerated at 0.3 V_air_/V_PBR_/min with filtered air (0.22 µm sterile air-venting filter, MillexFG50-Millipore) enriched with CO_2_ (7% V_CO2_/V_air_), which was injected on demand to keep a constant pH (7.5 ± 0.5) regulated by a pH sensor. At the end of the culture, the 10 L PBR was dismantled, and microalgae biomass was harvested by 7 min centrifugation of the cellular suspension at 6461 g in a SIGMA 2-16P centrifuge. Microalgae biomass was then washed twice with distilled water, oven dried during 24 h at 70 °C, homogenized, freeze-dried (CoolSafe freeze dryer, 100-9 PRO) and stored.

### 2.2. Adsorption Experiments

#### 2.2.1. Chemicals and Analytic Methods

Ibuprofen sodium (≥ 98%) was purchased from Fluka (Buchs, Switzerland) and salicylic acid (≥ 99%) was purchased from Sigma–Aldrich (Steinheim, Germany). The main properties of these pharmaceuticals are depicted in Table 1.

The target pharmaceuticals were analysed by high performance liquid chromatography (HPLC), using a Waters HPLC 600 equipped with a 2487 Dual λ Absorbance Detector, a phenomenex C18 column (5 μm, 110 Å, 250 × 4.6 mm), a Rheodyne injector and a 50 μL loop. The wavelengths of detection were 220 and 236 nm for ibuprofen and salicylic acid, respectively. The mobile phase was a mixture of methanol:water:orthophosphoric acid (75:25:0.3) for the analysis of ibuprofen and a mixture of acetonitrile:water:orthophosphoric acid (70:30:0.1) for the analysis of salicylic acid. The mobile phase mixtures were prepared using HPLC quality acetonitrile (CH_3_CN), which was purchased from LabScan, HPLC quality methanol (CH_3_OH), which was acquired from Sigma–Aldrich, orthophosphoric acid (H_3_PO_4_), which was purchased from Panreac, and ultrapure water obtained by a Millipore system. Before use, the mobile phase mixtures were filtered (0.45 µm pore size Millipore filters) and degasified (30 min under ultrasonication). For the chromatographic analytic quantification of pharmaceuticals concentration, four replicated injections were done under a 1 mL min^−1^ flow rate of the mobile phase, with precision ranging between 0.35 and 0.97 RSD(%) for ibuprofen and between 0.30 and 0.56 RSD(%) for salicylic acid. For each pharmaceutical, the limit of detection (LOD) and limit of quantification (LOQ) were determined on the basis of the signal-to-noise ratio using analytical response of 3 and 10 times of the background noise, respectively. The determined LOD/LOQ for ibuprofen and salicylic acid were 0.69/2.3 mg/L and 0.39/1.3 mg/L, respectively. Water samples analysed for the residual concentration of ibuprofen or salicylic acid after adsorption experiments were not subjected to any preconcentration or clean-up process before chromatographic analysis.

#### 2.2.2. Adsorption Kinetics and Equilibrium

Batch adsorption experiments on the adsorptive removal of ibuprofen and salicylic acid by biomass of *Scenedesmus obliquus* were carried out. Before its use as biosorbent, freeze-dried microalgae biomass was grinded and homogenized. Under identical conditions and for comparison purposes, adsorption reference experiments were also carried out using a commercial activated carbon, namely Pulsorb WP260 (Chemviron Carbon, Feluy, Belgium), which was generously provided by Chemviron Carbon and whose physicochemical properties are depicted in Table S1. First, and for each of the target NSAIDs, adsorption kinetic experiments were done so to find out the adsorption equilibrium time (*t_eq_*) and the adsorption rate. Next, equilibrium experiments were run to determine the corresponding adsorption isotherm. All experiments were carried in a thermostatically regulated shaker, where 250 mL Erlenmeyer flasks containing a known mass of adsorbent together with 100 mL of pharmaceutical solution adjusted to pH 7, by the addition of NaOH, were shaken (250 rpm) at a constant temperature of 25 ± 2 °C. The mass of *Scenedesmus obliquus* used as adsorbent in kinetic experiments was 0.05 g, while different masses in the range 0.05–0.5 g were used for equilibrium experiments. The pH of adsorption experiments (pH 7) was selected because it is a typical value for domestic wastewaters [49] and within the expected range after the secondary treatment (6.5–7.5) [50]. All experiments were done in triplicate and with an initial pharmaceutical concentration of 100 ± 1 mg L^−1^. In parallel with adsorption experiments, triplicate controls (pharmaceutical solution with an initial concentration of 100 ± 1 mg L^−1^) and triplicate blanks (known mass of adsorbent in ultrapure water), were also run. 

In the kinetic experiments, Erlenmeyer flasks were progressively withdrawn from the shaker after pre-set time intervals. Then, from each flask, three aliquots were collected, filtered through Millipore membranes (45 µm pore size) and analysed for the concentration of the target pharmaceutical, either ibuprofen or salicylic acid, as described in Section 2.2.1 For each of the pharmaceuticals, the adsorbed concentration onto the corresponding adsorbent at any time, *q_t_* (mg g^−1^), was calculated by the following mass balance relationship (Equation (1)):

(1) $$q_{t}=\left(C_{0}-C_{t}\right)\frac{V}{W}$$

where *C_t_* (mg L^−1^) is the experimental liquid-phase concentration of pharmaceutical at a time *t* (min), *C_0_* (mg L^−1^) is the average concentration of pharmaceutical in the corresponding control, *V* is the solution volume (L) and *W* is the adsorbent mass (g).

In the equilibrium experiments, Erlenmeyer flasks containing the target pharmaceutical solution together with either microalgae biomass or activated carbon were shaken during the *t_eq_* inferred from kinetic experiments. At that moment, three aliquots were withdrawn from each flask, filtered through Millipore membranes (45 µm pore size) and chromatographically analysed for determining the experimental equilibrium liquid-phase concentration of pharmaceutical, *C_e_* (mg L^−1^)). Then, the equilibrium adsorbed concentration of pharmaceutical onto the corresponding adsorbent, *q_e_* (mg g^−1^), was calculated by the mass balance relationship displayed in Equation (2):

(2) $$q_{e}=\left(C_{0}-C_{e}\right)\frac{V}{W}$$

where *C_e_* (mg L^−1^) is the experimental liquid-phase concentration of pharmaceutical at equilibrium, *C_0_* (mg L^−1^) is the average concentration of pharmaceutical in the corresponding control, *V* is the solution volume (L) and *W* is the adsorbent mass (g).

#### 2.2.3. Modelling of Kinetic and Equilibrium Results

Fittings of the obtained kinetic results to the pseudo-first order [51] and the pseudo-second order [52] equations, which are represented by Equations (3) and (4), respectively, were determined.

(3) $$q_{t}=q_{e}(1-e^{-k_{1}t\;})$$

(4) $$q_{t}=\frac{q_{e}^{2}k_{2}t}{1+q_{e}k_{2}t}$$

where *q_t_* (mg g^−1^) is the adsorbed concentration onto the corresponding adsorbent at any time, *q_e_* (mg g^−1^) is the adsorbed concentration of pharmaceutical onto the corresponding adsorbent at the equilibrium, *k_1_* (min^−1^) and *k_2_* (mg^−1^ g min^−1^) are the pseudo-first and the pseudo-second order rate constants, respectively, and *t* (min) is the time.

Regarding the experimental adsorption equilibrium results, fittings to the Freundlich isotherm [53] and the Langmuir isotherm [54], respectively represented by Equations (5) and (6), were determined.

(5) $$q_{e}=K_{F}C_{e}^{1/n}$$

(6) $$q_{e}=\frac{Q_{m}K_{L}C_{e}}{1+K_{L}C_{e}}$$

where *q_e_* (mg g^−1^) is the adsorbed concentration of pharmaceutical onto the corresponding adsorbent at the equilibrium, *C_e_* (mg L^−1^) is the liquid-phase concentration of pharmaceutical at equilibrium, *K_F_* is the Freundlich equilibrium constant (mg g^−1^ (mg L^−1^) ^−1/n^); *n* the degree of nonlinearity; *Q_m_* the Langmuir maximum adsorption capacity (mg g^−1^) and *K_L_* (L mg^−1^) is the Langmuir equilibrium constant.

#### 2.2.4. Thermodynamics of Adsorption

Changes of Gibb’s free energy (Δ*G^°^*, kJ mol^−1^), enthalpy (Δ*H^°^*, kJ mol^−1^) and entropy (Δ*S^°^*, kJ mol^−1^ K^−1^) in the adsorption of salicylic acid and ibuprofen onto *Scenedesmus obliquus* biomass were calculated as next depicted.

The Van’t Hoff equation measures the changes in the thermodynamic equilibrium constant (*K_e_*) with variations of the temperature, as for Equation (7).

(7) $$\Delta G{}^{\circ}=-RT\;lnK_{e}^{o}$$

where Δ*G^°^* is the Gibb’s free energy (kJ mol^−1^), *R* is the universal gas constant (8.314 J K^−1^ mol^−1^), *T* is the absolute temperature (Kelvin), and *K_e_^°^*, which is dimensionless, was obtained by the following equation, as recommended by Lima *et al.* [55]:

(8) $$K_{e}^{o}=\frac{1000\;K_{L}\;MW\;\left[adsorbate\right]{}^{\circ}}{\gamma }$$

where *γ* is the coefficient of activity (dimensionless), [adsorbate] is the standard concentration of the adsorbate (1 mol L^−1^) and *K_L_* (L mg^−1^) is the Langmuir equilibrium constant, as for the best fitting model. In order to *K_L_* (L mg^−1^) become dimensionless, it was multiplied by 1000, to convert L mg^−1^ into L g^−1^, subsequently making the multiplication of this result by *MW*, which is the molecular weight of the adsorbate (g mol^−1^), and by [*adsorbate*]^°^, which is the unitary standard concentration of the adsorbate (1 mol L^−1^). For this calculation, it is assumed a diluted adsorbate solution so γ is unitary.

Considering the third principle of thermodynamics:

(9) ΔG°=ΔH°−TΔS°

where Δ*G^°^* is the change in Gibb’s free energy (kJ mol^−1^), Δ*H^°^* is the change in enthalpy (kJ mol^−1^), Δ*S^°^* is the change in entropy (kJ mol^−1^ K^−1^) and *T* is the absolute temperature (K).

The combination of Equations (7) and (9), gives:

(10) $$lnK_{e}^{o}=\frac{-\Delta H{}^{\circ}}{R}\;\frac{1}{T}+\frac{\Delta S{}^{\circ}}{R}$$

where *K^°^_e_* is the standard thermodynamic equilibrium constant (dimensionless), *R* is the universal gas constant (8.314 J K^−1^ mol^−1^), *T* is the absolute temperature (K), Δ*H^°^* is the change in enthalpy (kJ mol^−1^) and Δ*S^°^* is the change in entropy (kJ mol^−1^ K^−1^).

Thus, in the linear plot of Ln (*K_e_^°^*) versus 1/T, Δ*S^°^* may be obtained from the intercept and Δ*H^°^* from the slope. For determining such a linear plot, at least three different *K_e_^°^* are necessary. Therefore, apart from the equilibrium isotherm obtained at 25 ± 2 °C for the adsorption of salicylic acid and ibuprofen onto microalgae biomass, isotherms were also determined at 15 ± 2 and 35 ± 2 °C by following the procedure described in Section 2.2.2. 

### 2.3. Microalgae Biomass Characterization

Point of zero charge (pH_PZC_), Fourier transmittance infrared (FT-IR) spectra, simultaneous thermal analysis (STA) curves and micrographs by scanning electron microscopy with energy dispersive spectroscopy (SEM/EDS) were used to characterize *Scenedesmus obliquus* biomass before and after salicylic acid and ibuprofen biosorption. For the characterization, freeze-dried microalgae biomass was dried in an oven (Selecta P, 2000208, E.U.) at 70 °C for 24 h and cooled in a desiccator, ground with a mortar and a pestle and then stored at room temperature. Taking into account that the considered pharmaceuticals are thermally stable at 70 °C (Table 1), the same procedure was applied to biomass to be characterized after its use for the adsorption of salicylic acid and ibuprofen.

#### 2.3.1. Point of Zero Charge (pH_PZC_) Determination

The point of zero charge was determined according to the method proposed by Rivera-Utrilla [56]. A mass of 50 mg of alga and 50 mL of 0.1 mol L^−1^ NaCl (VWR International, purity: 99.9%) were mixed in Erlenmeyer flasks. The pH of each suspension was adjusted to an initial pH value (pH_initial_) in the range 2 to 11, using 0.01 mol L^−1^ NaOH (Labkem, purity: >99.0%) or 1 mol L^−1^ HCl (Honeywell Fluka™, 37% wt). Samples were stirred during 24 h and the final pH (pH_final_) was recorded using a pH-meter Consort C861. The pH_final_ was plotted against pH_initial_, the point at which the curve crossed the line pH_final_ = pH_initial_ was taken as the PZC (pH_pzc_).

#### 2.3.2. Fourier Transform Infrared (FT-IR) Analysis

The infrared spectra of the samples diluted with KBr and pressed (press PanReac, PAI for IR spectroscopy) (0.5 mg of ground sample with 200 mg of KBr) were recorded in the range of 4000–500 cm^−1^ with resolution of 2 cm^−1^ (200 scans) (Nicolet 6700 FT-IR, Thermo Scientific, MCT/A detector). Spectra were acquired and processed with the OMNIC Software Version *8.3.103* [57].

#### 2.3.3. Simultaneous Thermal Analysis (STA)

The simultaneous thermal analysis was carried out in a Netzsch STA 449 F3 Jupiter thermal analyser. The experimental procedure described by Valenzuela and Bernalte García [58] and Ferreira et al. [59] was followed for the proximate analysis of *Scenedesmus obliquus* biomass before and after the adsorption of salicylic acid and ibuprofen. Briefly, samples (mass of approximately 10 mg) were heated at a constant rate of 10.0 K min^−1^ from room temperature to 950 °C. After 7 min of isothermal treatment at 950 °C under nitrogen atmosphere at a flow of 50 mL min^−1^, the nitrogen was changed by air at a flow of 50 mL min^−1^ extending the treatment until final stabilization of final weight. Under this procedure, the mass loss observed at 100 °C is attributed to moisture, the mass loss registered from the end of the first step up to the switching of the carrier gas corresponds to the volatile matter and the mass loss comprised between the introduction of the air flow and the stabilization of the weight is attributed to the fixed carbon content [59]. Thermogravimetry (TG), derivate thermogravimetry (DTG), differential scanning calorimetry (DSC) and derivate differential scanning calorimetry (DDSC) curves were acquired and processed with the software NETZSCH Proteus Thermal analysis version 5.2.1.

#### 2.3.4. Scanning Electron Microscopy with Energy Dispersive Spectroscopy (SEM/EDS) 

The images of scanning electron microscopy with energy dispersive spectroscopy were obtained using a high-resolution scanning electron microscope with X-Ray microanalysis (JEOL JSM 6301F/ Oxford INCA Energy 350) [60]. Samples were coated with an Au/Pd thin film, by sputtering, using the SPI Module Sputter Coater equipment, for 100 s with a 15-mA current. The micrographs were generated by secondary electrons (SE), with a × 2000 magnification, an accelerating voltage of 15 kV and a working distance (WD) of 15 mm.

## 3. Results and Discussion

### 3.1. Adsorption Results

#### 3.1.1. Adsorption Kinetic and Equilibrium 

As confirmed by the controls and blanks run in parallel with adsorption experiments, pharmaceuticals adsorption on glass and any other loss were negligible, so the decrease in salicylic acid and ibuprofen concentrations during these experiments is ascribable just to the adsorptive removal onto microalgae biomass or onto the commercial activated carbon used as reference (Pulsorb WP260).

**Figure 1:**
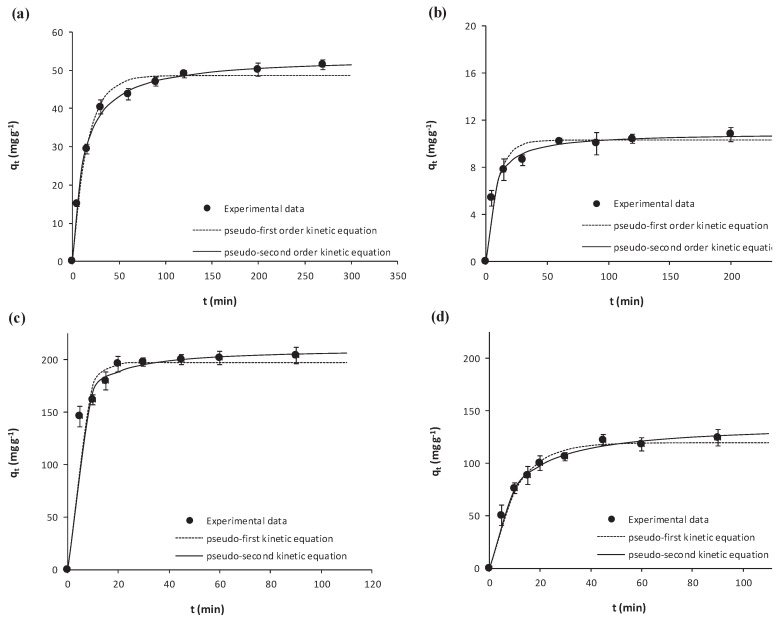
Kinetic results on the adsorption of (**a**) salicylic acid and (**b**) ibuprofen onto *Scenedesmus obliquus* biomass; (**c**) salicylic acid and (**d**) ibuprofen onto commercial activated carbon used as reference.

Kinetic results on the adsorption of salicylic acid and ibuprofen onto microalgae biomass or onto Pulsorb WP260 throughout time are depicted in Figure 1, together with fittings to the pseudo- first and the pseudo-second order equations (Equations (3) and (4), respectively). Error bars in Figure 1 stand for the corresponding standard deviations (N = 3). The axes’ scale has been adjusted for a better visualization of results. As may be seen, for each of the target NSAIDs, the adsorbed concentration increased throughout contact time with the corresponding adsorbent until becoming stable at the equilibrium. 

Figure 1 evidences that both the pseudo-first and pseudo-second order kinetic equations reasonably describe experimental results, with slightly better fittings to the pseudo-second order equation. Kinetic parameters derived from the fittings to the models are depicted in Table 2 together with the corresponding determination coefficient (*r*^2^) and standard deviation of the residuals (*S_y.x_*), which confirm that the pseudo-second order equation generally provided better fittings (*r*^2^ > 0.99 and lower *S_y.x_*). According to the fitted parameters, the largest kinetic constant *k_2_* was that of ibuprofen adsorption onto *Scenedesmus obliquus* biomass, with the rest being comparable. In any case, and in order to ensure that equilibrium was attained, a contact time of 150 min was defined for all the equilibrium experiments.

**Figure 2:**
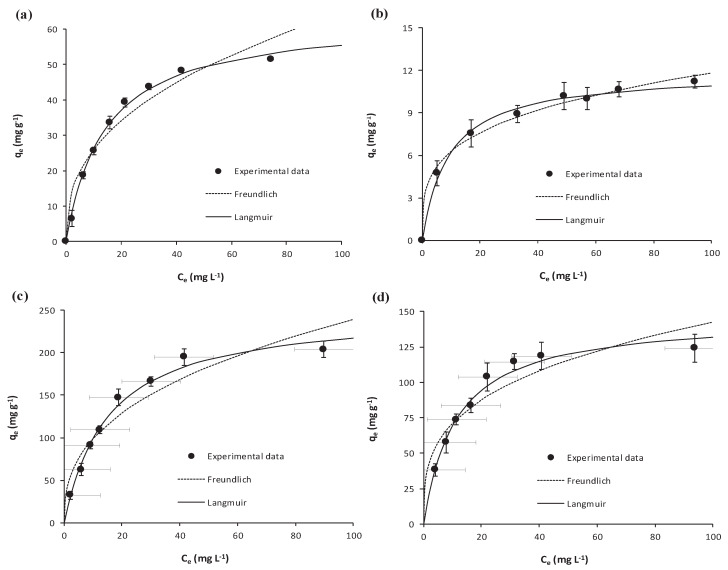
Equilibrium results on the adsorption of (**a**) salicylic acid and (**b**) ibuprofen onto *Scenedesmus obliquus* biomass; (**c**) salicylic acid and (**d**) ibuprofen onto commercial activated carbon used as reference.

Equilibrium isotherms determined for the adsorption of salicylic acid and ibuprofen onto *Scenedesmus obliquus* biomass and onto the activated carbon are shown in Figure 2 together with the fittings to the Freundlich and Langmuir isotherms. Error bars stand for the corresponding standard deviations (N = 3). The axes’ scale has been adjusted for a better visualization of results. For each of the isotherm models, fitted parameters are depicted in Table 2.

As shown in Figure 2, at the equilibrium, the adsorptive removal of both NSAIDs is larger onto the commercial activated carbon than onto microalgae biomass. Furthermore, both the activated carbon and microalgae biomass, displayed a larger adsorption for salicylic acid than for ibuprofen, which can be associated with the lower solubility of salicylic acid when compared with ibuprofen (Table 1).

With respect to the fittings, experimental results are better described by the Langmuir isotherm than by the Freundlich one, which is confirmed by the respective higher *r*^2^ and lower *S_y.x_* in Table 2. Indeed, *Q_max_* values from the Langmuir fittings confirm that the adsorption capacity of activated carbon (*Q_max_* = 250 mg g^−1^ for salicylic acid and *Q_max_* = 147 mg g^−1^ for ibuprofen) is larger than that of *Scenedesmus obliquus* biomass (*Q_max_* = 63 mg g^−1^ for salicylic acid and *Q_max_* = 12 mg g^−1^ for ibuprofen), which must be related to the highly porous structure of the carbon. It must be highlighted that the here determined *Q_max_* for ibuprofen adsorption onto microalgae biomass is higher than that determined for the adsorption of this pharmaceutical onto *Phaeodactylum tricornutum* (*Q_max_* = 4 mg g^−1^) [32], and is also higher than that of oxytetracycline onto *Phaeodactylum tricornutum* (*Q_max_* = 5 mg g^−1^) [21]. To the best of our knowledge, there are not published results on the adsorption of salicylic acid onto microalgae biomass. In any case, the here obtained *Q_max_* is larger than that determined for the adsorption of acetaminophen onto *Synechocystis sp.* (*Q_max_* = 52 mg g^−1^) [41] and quite larger than for the adsorption of diclofenac onto *Scenedesmus obliquus* biomass (*Q_max_* = 28 mg g^−1^) [31]. With respect to other materials used for the biosorption of salicylic acid or ibuprofen from water, Table S2 (Supplementary Information) [32,61,62,63,64] depicts some recently published *Q_max_* and shows that the here determined values for *Scenedesmus obliquus* biomass are within the range of values in the literature.

#### 3.1.2. Thermodynamics of Adsorption 

**Figure 3:**
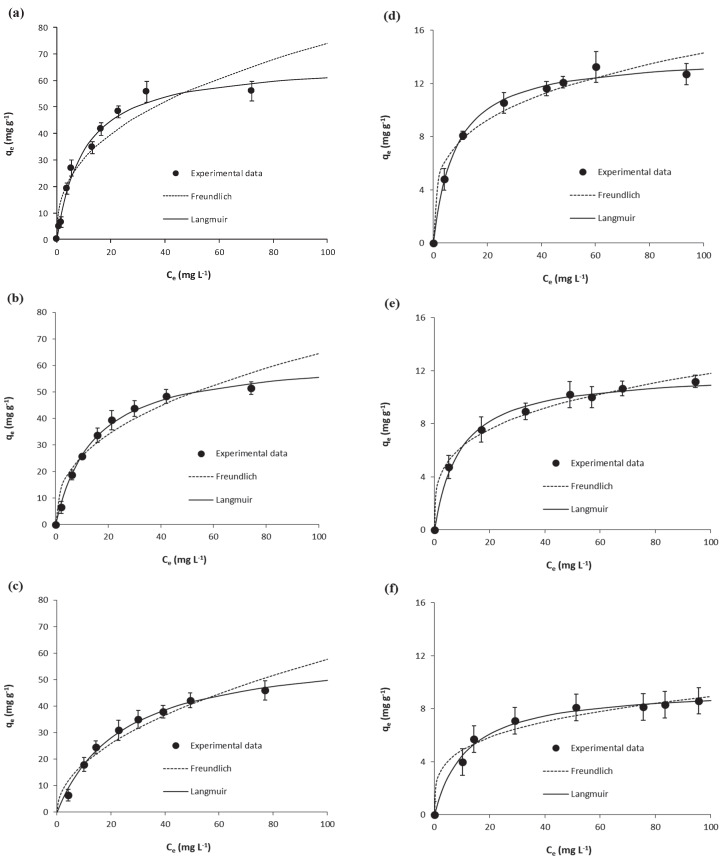
Equilibrium results on the adsorption of salicylic acid (at (**a**) 15 °C, (**b**) 25 °C and (**c**) 35 °C) and ibuprofen (at (**d**) 15 °C, (**e**) 25 °C and (**f**) 35 °C) onto *Scenedesmus obliquus* biomass.

Equilibrium results on the adsorption of salicylic acid and ibuprofen onto *Scenedesmus obliquus* biomass at the different temperatures considered in this work are shown in Figure 3 together with fittings to the Langmuir and Freundlich isotherms. Parameters determined from these fittings are depicted in Table S3 (Supplementary Information). As may be seen, Langmuir described the experimental results (*r^2^* ≥ 0.99) and the determined *K_L_* values were used in Equation (8) for the calculation of the corresponding *K_e_^°^*, as described in Section 2.2.4. 

Thermodynamic parameters are presented in Table 3. The negative *∆G^°^* values point to the feasibility of the adsorption of salicylic acid and ibuprofen on microalgae biomass, which is thermodynamically spontaneous. The negative ∆*H*^°^ values, namely −32 and −13 kJ mol^−1^ for the adsorption of salicylic acid and ibuprofen, respectively, indicate that adsorption onto microalgae biomass was exothermic. 

The type of adsorptive interaction can be classified, to a certain extent, by the magnitude of ∆*H*^°^. In this sense, energy related to chemical bond forces is usually >80 kJ mol^−1^, while physical sorption by hydrogen bonding is usually <30 kJ mol^−1^, by van der Waals forces is 4–10 kJ mol^−1^, by hydrophobic bond forces about 5 kJ mol^−1^, by coordination exchange about 40 kJ mol^−1^ and by dipole bond forces 2–29 kJ mol^−1^ [65]. Therefore, the here obtained values are consistent with interaction by hydrogen bond forces between microalgae biomass and both salicylic acid and ibuprofen. As for the entropy changes, although the values were quite low, the negative and positive *∆S^°^* obtained for the adsorption of salicylic acid and ibuprofen, respectively, revealed the respective decrease and increase in randomness at the aqueous–solid interface during the adsorption of these pharmaceuticals onto microalgae biomass.

### 3.2. Microalgae Biomass Characterization

#### 3.2.1. pH_PZC_

**Figure 4:**
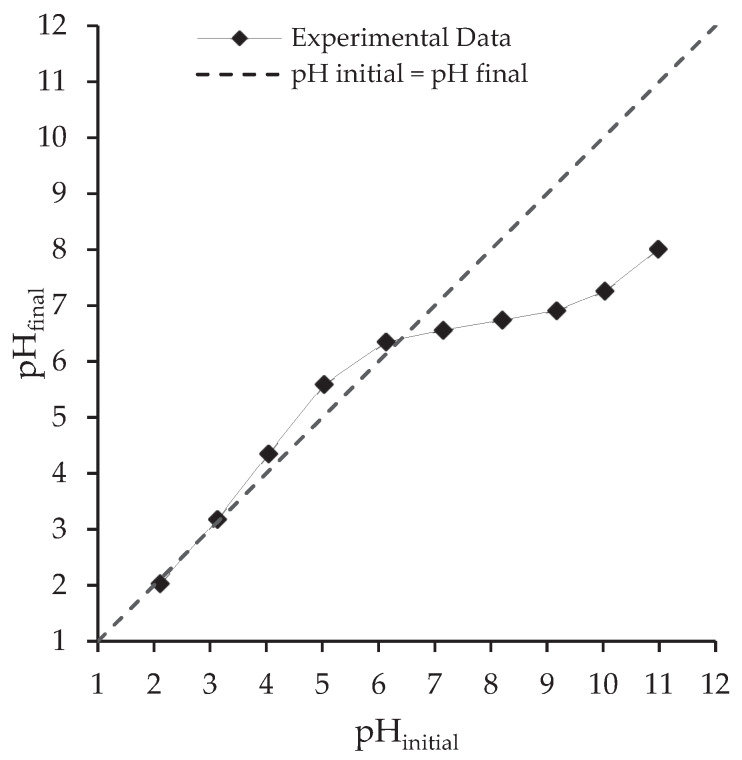
Point of zero charge (pH_PZC_) determination of *Scenedesmus obliquus* biomass.

The sorption of compounds is strongly influenced by the system pH. The microalgae surface functional groups (Section 3.2.2.) can be positively or negatively charged depending on their *p*Ka values. These groups are subject to protonation or deprotonation depending on the pH value of the solution. The pH_PZC_ influences the biosorption of charged species. The pH_PZC_ here determined for *Scenedesmus obliquus* biomass was 6.4 (Figure 4). For pH values below pH_PZC_, the surface charge of microalgae biomass is positive so negative species biosorption is favoured; whereas for pH values higher than pH_PZC_, the surface charge is negative and positive species biosorption is favoured [66]. The pH can also impact the charge of the species in solution [67,68]. As shown in Table 1, the *p*Ka_1_ (carboxylic group) and *p*Ka_2_ (phenol group) values of salicylic acid are 3.0 and 13.6, respectively and the *p*Ka (carboxylic group) value of ibuprofen is 4.9. In the case of salicylic acid, diprotic species (neutral) are dominant (about 90%) at pH values below 2, monoprotic species (negative) are dominant at pH values between 4 and 13 and completely dissociated species (negative) are dominant (about 70%) at pH values above 13 [69] (see Figure S1a (Supplementary Information) [69,70]). The carboxylic group of ibuprofen is not charged at low solution pH (equal or below to *p*Ka). However, as the solution pH increases from 3 to 7, the carboxylic group begins to dissociate and almost all ibuprofen molecules are negatively charged when the solution pH is above 7 [70,71] (see Figure S1b (Supplementary Information) [69,70]). Thus, at the pH of adsorption experiments (pH 7), electrostatic repulsive interactions between the negatively charged surface of *Scenesdesmus obliquus* and the negatively charged ibuprofen and salicylic acid and ibuprofen species cannot be disregarded.

#### 3.2.2. FT-IR

**Figure 5:**
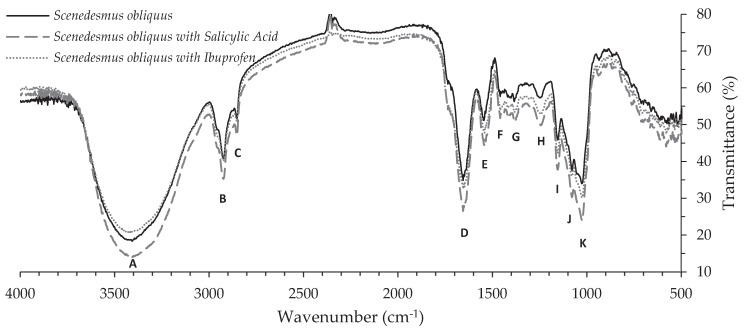
Fourier transform infrared spectra of *Scenedesmus obliquus* biomass before and after salicylic acid and ibuprofen biosorption.

The functional groups present on microalgae biomass surface play an important role in the biosorption process. FT-IR spectra from *Scenedesmus obliquus* biomass before and after salicylic acid and ibuprofen biosorption are shown in Figure 5 and the assignation of bands in Table S4. Eleven main bands can be identified in the wavenumber range recorded. The three spectra are very similar, exhibiting slight variations between them; as such, each band was identified by a wavenumber range. The 4000–2500 cm^−1^ region corresponds to the stretching vibrations of bonds containing hydrogen atoms. The single broad band at about 3408–3423 cm^−1^ (band A) is assigned to O-H stretching vibrations (associated) of water or hydroxyl radicals of polysaccharides [72,73,74,75], and to N-H stretching vibrations of proteins (amide A) [72,73,75]. Below 3000 cm^−1^, absorption bands at about 2924 cm^−1^ and 2853 cm^−1^ (bands B and C, respectively) are assigned to C-H stretching vibrations, more specifically to CH_2_ asymmetric and symmetric stretching vibrations of lipids, respectively [72,73,74,75,76,77,78]. The range 2000–1500 cm^−1^ is the region for double bonds. The absorption band at about 1655 cm^−1^ (band D) is in the range of 1900–1650 cm^−1^ where carbonyl group usually shows a strong band. This band corresponds to C=O stretching vibration of amides I, which is associated with proteins [72,76,78]. The band at about 1541–1545 cm^−1^ (band E) is due to N-H deformation of amides II and to C-N deformation of proteins [72,73,74,75,76,77,78]. Bands in 1500–1300 cm^−1^ region provide the information on the deformation vibrations of C-H bonds. Bands F and G at about 1458 and 1383–1384 cm^−1^, respectively, are associated with CH_3_ asymmetric deformations of lipids [72,74,75,77] and proteins, and also to C-O symmetric stretching vibrations of carboxylic groups [72,74,75,76,77,78]. The branching in the band at about 1380 cm^−1^ indicates that two or three methyl groups may be connected to the same carbon atom. Below 1300 cm^−1^ appear the bands from C-O-C bonds of carboxylates. Bands at about 1153–1154 cm^−1^, 1078–1079 cm^−1^ and 1025–1026 cm^−1^ (bands I, J and K, respectively) are assigned to C-O-C stretching vibrations of polysaccharides from carbohydrates [73,74,75,76,77,78]. These bands are also assigned to C-O asymmetric stretching vibrations of starch and complex sugar ring modes [72,73,74,75,77] and to Si-O stretching vibrations of silicate frustules [76,78]. Bands J and K are also assigned to P=O asymmetric stretching vibrations of phosphodiester backbone from nucleic acids, as well as the band at about 1241–1246 cm^−1^ (band H) [72,73,74,75,76,78]. The FT-IR spectrum of *Scenedesmus obliquus* is mostly the same as the determined by Ferreira et al. [79] for this microalgae species but different from those observed by these authors for *Chlorella vulgaris* or *Nannochloropsis oculata*. As shown in Figure 5 and Table S4, the spectra show just small punctual shifts between absorption bands determined for microalgae biomass before and after salicylic acid and ibuprofen biosorption. Similarly, Santaeufemia et al. [21] did not found differences in the FT-IR spectra of *Phaeodactylum tricornutum* biomass before and after the adsorption of triclosan, which related to the absence of chemical bonds during adsorption. Furthermore, Cardoso et al. [65] found that the wavenumbers of the vibrational bands in *Spirulina platensis* biomass were practically the same before and after the adsorption of Reactive Red 120, concluding that the interaction adsorbent–adsorbate presented low energy. Differently, Ali et al. [63] found that the adsorption of tramadol onto alkaline modified biomass of *Scenedesmus obliquus* resulted not only in some slight shifts and intensity decrease, but also in the disappearance of bands 3326 and 1419 cm^−1^. These authors pointed to the possible involvement of O-H and C-O functional groups in the adsorption of tramadol by modified algal biomass [63]. Likewise, significant changes were observed by Koshua et al. [80] in the FT-IR spectra of *Scenedesmus quadricauda* and *Chlorella vulgaris* biomasses after malachite green adsorption, which were ascribed to the attachment of this dye on –OH and –NH groups. Therefore, in the present work, it may be inferred that adsorption of salicylic acid and ibuprofen onto *Scenedesmus obliquus* biomass was mostly physical and, although some low energy interactions may have occurred with –OH and –NH groups, the importance of chemical bonding with biomass surface was minor. Indeed, physical adsorption inferred by FT-IR results is consistent with the relative low energy involved in the adsorption of the considered NSAIDs, as for the low ∆*H*^°^ displayed in Table 3. The bands at about 1589–1591, 1487 and 1458 cm^−1^, characteristic of C-C stretching vibrations of benzene ring from salicylic acid, are not present in the alga’s spectra after biosorption of this pharmaceutical [81]. Likewise, the high intense band at about 1720 cm^−1^ characteristic of C=O stretching vibration of ibuprofen is also not present [82,83]. This may be related to the relatively low proportion of both adsorbed pharmaceuticals in comparison to the mass of microalgae and/or to the overlapping of other stronger bands in the same wavenumbers. 

#### 3.2.3. STA

**Figure 6:**
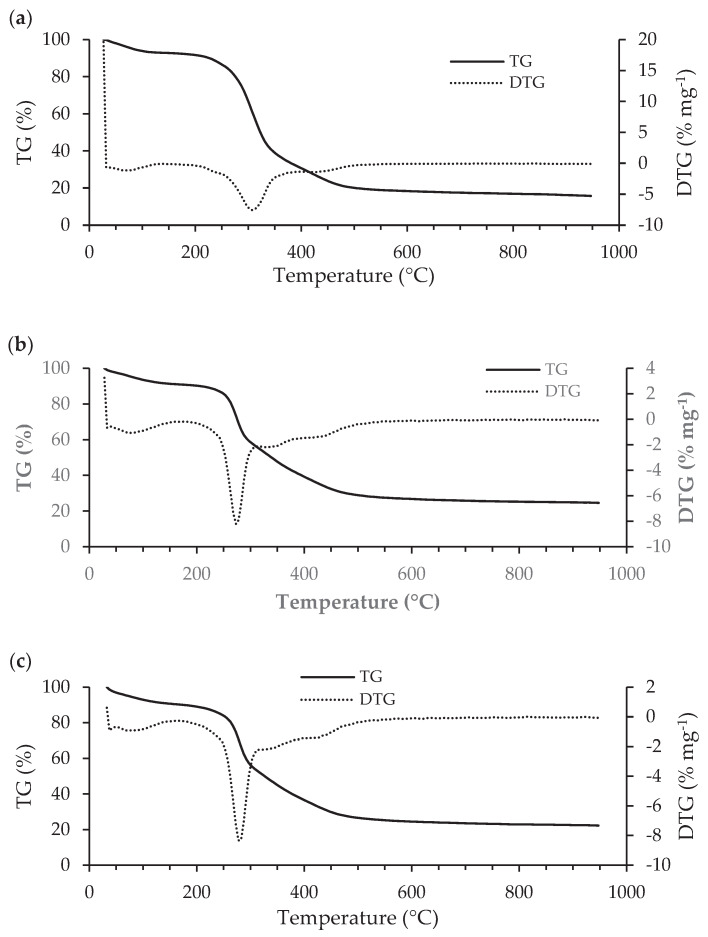
Thermogravimetry (TG) together with derivate thermogravimetry (DTG) curves of *Scenedesmus obliquus* biomass before biosorption (**a**); and after salicylic (**b**) or ibuprofen (**c**) biosorption.

Simultaneous thermal analysis (STA) allows identifying mass losses and thermal transitions occurred during the decomposition of *Scenesdesmus obliquus* biomass before and after pharmaceuticals biosorption through the interpretation of TG/DTG curves, which are shown in Figure 6 (TG/DTG curves over time are also shown in Figure S2 (Supplementary Information)) and DSC/DDSC curves, which are depicted Figure S3. The TG/DTG and DSC/DDSC characteristic parameters are summarized in Table 4 and Table S5, respectively. The initial temperature (T_i_) and the final temperature (T_f_), which are, respectively, the lowest temperatures at which the start and the end of a mass variation can be detected in TG/DTG curves, were used to determine the moisture, volatile matter, fixed carbon and ash contents depicted in Table 5. The maximum temperature (T_TG,max_) is the temperature at which the maximum mass loss rate (DTG_max_) is reached. The here obtained results allow to observe a similar thermal degradation profile for the three samples, namely microalgae biomass before and after biosorption of salicylic acid or ibuprofen. In good agreement with literature [84,85], the thermal degradation of microalgae biomass before and after adsorption can be divided into two main mass loss steps and three DSC peaks. All DSC curves in Figure S3 show a short transient period of instability at the start of the run, which corresponds to the time required for energy to be transmitted to both sample and reference in order to produce the required heating rate [86]. Meanwhile, mass loss develops quite slowly from the initially horizontal part of the TG curves. 

In the first step of the process, the slight mass loss from room temperature to about 100 °C is attributed to water evaporation. The estimated moisture contents (Table 5) are very similar and below 8%, which certainly favoured an easy ignition and prevented a reduction of the heating. All T_DSC,max_ in Table S5 are lower than the boiling point of pure water and increase with increasing of moisture content. The endothermic event (peak A) present in all DSC curves (Figure S3) confirms that the mass loss is associated with samples’ dehydration. The respective peak characteristics depicted in Table 4 also allow to verify that T_TG,max_ increases with increasing moisture content, from *S. obliquus* to *S. obliquus* after ibuprofen biosorption. Contrarily, the ΔH values (Table S5) decrease with increasing moisture content. 

The second step corresponds to the main thermal degradation step of the process. The significant inflection observed in TG curves, from the 100 to about 550 °C, is attributed to devolatilization. The highest estimated volatile matter content and T_TG,max_ (Table 4) is observed for *S. obliquus*. The pharmaceuticals’ uptake reduced the volatile matter content and the T_TG,max_ of microalgae biomass similarly for both salicylic acid and ibuprofen. Due to the adsorption of pharmaceuticals, the loss of volatiles during pyrolysis, in percentage terms, was reduced. Contrarily, the fixed carbon increased, which reflects the losses under combustion, due to the contribution of thermal oxidation of adsorbed pharmaceuticals. Indeed, the T_TG,max_ values after adsorption are between the melting points of salicylic acid and ibuprofen (158 °C and 76 °C [45,46], respectively) and the T_TG,max_ value of *S. obliquus* before biosorption. The exothermic event (peak B) present in all DSC curves (Figure S3) confirms that mass loss is associated with the decomposition of biological molecules (carbohydrates, lipids, proteins and nucleic acids), as stated in FT-IR analysis. According to a previous study [87] photosynthetic pigments are degraded at 190 °C. Since *S. obliquus* is a green microalga, it can be assumed that photosynthetic pigments were also decomposed in this step. 

Although no significant peak is observed in DTG curves (Figure S3), from 550 to 950 °C under nitrogen atmosphere, the carbonaceous matter continuously decomposed at a very low rate, which is confirmed by the exothermic event (peak C) present in all DSC curves. As for B peak, the highest T_DSC,max_ (Table S5) for peak C is also observed for *S. obliquus*. In addition, as observed for B peak, the pharmaceuticals’ uptake reduced the T_DSC,max_ values similarly for both salicylic acid and ibuprofen. The ΔH values, in its turn, decrease significantly from *S. obliquus* to *S. obliquus* after ibuprofen biosorption. The mass loss verified between the introduction of the air flow and the stabilization of the weight is attributed to the fixed carbon content. The residue obtained at the end of the thermal process corresponds to the ashes. The ash contents (Table 5) obtained were very small (less than 3%), since microalgae grew under controlled laboratory conditions and the ash obtained necessarily came entirely from the biomass itself [88]. Fixed carbon content was determined by difference and represents the solid carbon in the biomass that remains after devolatilization. The obtained contents are between 13% and 22% and are inversely proportional to the volatile matter contents [88].

#### 3.2.4. SEM/EDS

**Figure 7:**
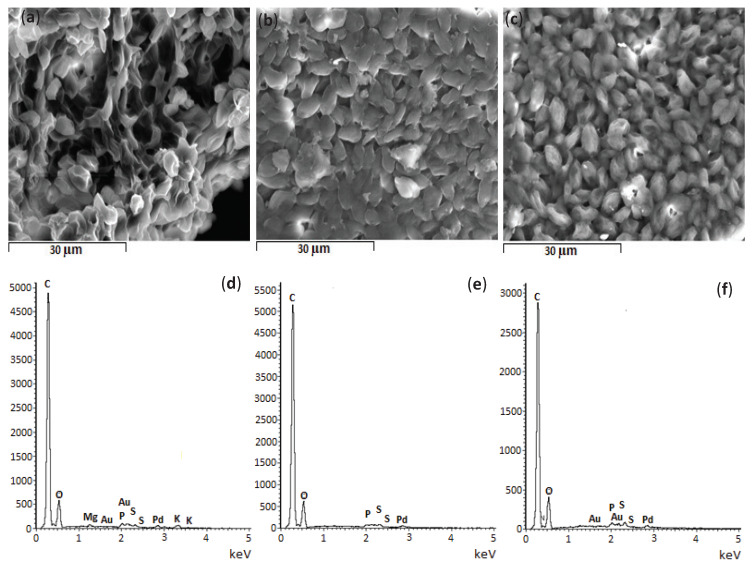
Scanning electron micrographs and energy dispersive spectroscopy graphs of *Scenedesmus obliquus* biomass (**a**,**d**) before and (**b**,**e**) after salicylic acid and (**c**,**f**) ibuprofen biosorption (SE; × 2000; 15 kV; WD = 15 mm).

The morphology and the elemental surface composition of *Scenesdesmus obliquus* surface was analysed by SEM coupled with EDS before and after salicylic acid and ibuprofen biosorption. The respective results are shown in Figure 7. No significant changes are observed in the surface morphology after pharmaceuticals uptake since microalgae cells kept their fusiform-elliptical shape [89]. The main difference seems to be the decrease in intercellular spaces, filled by the sorbed pharmaceuticals. 

Regarding the EDS analysis, it should be noted that spectra were obtained from single points and, as such, it is difficult to ensure the representativeness of the areas selected in the EDS analysis. The depth of EDS analysis was 15 mm, part of this depth corresponds to the Au/Pd film, and so pharmaceutical molecules sorbed at a depth greater than 15 mm were not detected. On the other hand, the peaks of gold (Au) and palladium (Pd) are associated with the coating film. Furthermore, the EDS results reveal the presence of other different chemical elements. Before pharmaceuticals’ uptake, carbon (C) and oxygen (O) are the predominant chemical elements in *S. obliquus* biomass, while other minerals (magnesium (Mg), sulphur (S), phosphorus (P) and potassium (K)) were detected at trace levels. This is also true after adsorption of salicylic acid and ibuprofen, although Mg and K were not detected after these pharmaceuticals’ uptake.

### 3.3. Final Remarks and Future Work

In this work, *Scenedesmus obliquus* residual biomass from microalgae culture was used for the adsorptive removal of two NSAIDs, namely salicylic acid and ibuprofen. The adsorption performance of such a biomass, which was thermodynamically favourable and exothermic, was comparable to that of a commercial activated carbon in kinetic terms but attained a relatively lower capacity. Nonetheless, the anionic state of the pharmaceuticals here considered at the pH of adsorption experiments (pH 7) may have reduced their adsorption on the negatively charged surface of microalgae biomass due to electrostatic repulsive interactions. It has already been shown that the adsorption of cationic pharmaceuticals would be more favourable [37,90]. In any case, it must be taken into account that commercial activated carbon is produced through chemical or physical activation and involves a pyrolytic thermal treatment of a non-renewable precursor, while in this work, microalgae biomass, which is renewable, was not subjected to any chemical modification or pyrolysis treatment. Besides the fact of most of the microalgae cell surface receptors for contaminants adsorption remain viable even after the cells have died [91], the use of dead biomass as adsorbent material holds some advantages related to the absence of living requirements and the associated costs. On the other hand, biosorption of pharmaceuticals by dead microalgae biomass is a passive nonmetabolic process that, therefore, does not result in the generation of metabolites or transformation products, which is an important factor for the choice of treatments aiming the removal of pharmaceuticals. As conventional adsorbents used for water treatment, such as activated carbon, microalgae biomass is not selective, meaning that, under the presence of other contaminants, binding sites may become saturated with nontarget contaminants. Therefore, adsorption processes for the removal of pharmaceuticals are used at the tertiary stage of wastewater treatment, under reduced competition by other types of contaminants, which were already removed in the first and secondary treatments. The adsorptive performance of microalgae biomass as tertiary treatment for the removal of pharmaceuticals needs to be further researched to assess its possible implementation at WWTPs. How the different pharmaceuticals present in wastewater interact with each other and affect their respective removal by microalgae biomass is another issue to investigate. Indeed, undertaking studies on pharmaceuticals removal under realistic conditions, assessing the effect of the pH and of the different components of complex matrices, is necessary. Moreover, different microalgae may have different adsorptive performance [31] so the characterization of biomass from different strains and the utilization of biomasses from assorted microalgae strains for the removal of mixed pharmaceuticals are to be explored. In order to select the most convenient species to be applied in wastewater treatments, information about the release of organic compounds during biosorption by microalgae biomass is essential and should be assessed. Furthermore, the after-use separation of microalgae from treated water is a challenge that needs to be addressed. Instead, the immobilization of microalgae biomass and the implementation of fixed-bed microalgae adsorbers [92] may be a more feasible choice. After-use, with the aid of a suitable desorbing agent, the adsorbed pharmaceuticals can be desorbed, and microalgae biomass can be reused [37]. Still, the effects of such a desorption treatment in the performance of reused biomass and the environmental and economic costs associated with the employment of chemical desorbing agents should be further assessed. Regeneration and reutilization capacities are not essential features of residual microalgae biomass, which is a low-cost adsorbent. Alternatively, energetic valorisation of microalgae biomass after adsorption saturation may be a more interesting option to consider.

Microalgae are photosynthetic microorganisms that are considered promising feedstock for the production of biofuels. On the other hand, microalgae cultivation offers interesting features such as the sequestration of CO_2_ from atmosphere or flue gases and the possibility of using wastewater as a low-cost growth media, simultaneously providing cost-benefits and enhancing sustainability of wastewater treatment. Indeed, microalgae-based wastewater treatment has been said to be the safest, promising, and most efficient alternative, replacing conventional treatment methods due to microalgae large availability, nutrient consumption ability and diverse applications of the biomass produced [93]. Moreover, integrating wastewater treatment and biorefinery may substantially contribute to circular economy [94]. In this sense, the utilization of residual microalgae biomass as adsorbent is a way to further exploit the potential of microalgae and minimize waste production. However, it is essential to carry out studies on such utilization for the removal of pharmaceuticals, since these are emerging contaminants whose discharge will probably become regulated in the near future.

## 4. Conclusions

The adsorptive performance of *Scenedesmus obliquus* biomass in the removal of salicylic acid and ibuprofen from water was assessed. Furthermore, microalgae biomass was characterized before and after adsorption of these pharmaceuticals in order to relate the obtained results on their removal with biomass properties and to find out if adsorption resulted in any modification. Fittings of the kinetic adsorption results to the pseudo-second order equation indicated that adsorption rates onto microalgae biomass were similar to those onto a commercial activated carbon used as reference. Equilibrium results were described by the Langmuir isotherm model and the fitted maximum adsorption capacities (*Q_max_*) for salicylic acid and ibuprofen were respectively 60 and 12 mg g^−1^, lower than those determined for the commercial activated carbon (250 and 147 mg g^−1^, respectively). Although microalgae present lower capacities than activated carbon, they may be seen as an alternative sustainable adsorbent. Regarding thermodynamic parameters, they indicated that adsorption of the target NSAIDs onto microalgae biomass was favourable, spontaneous and exothermic. Moreover, no significant modifications were detected on the surface groups of microalgae biomass after the adsorption of the pharmaceuticals. This fact, together with the energy associated with the process, allowed us to infer that their removal mostly occurred by physical interaction. Results obtained in this work point to the utilization of nonmodified dead microalgae biomass as an alternative green adsorbent for the removal of pharmaceuticals, such utilization being compatible with other uses of microalgae and therefore in line with circular economy and biorefinery principles.

## Figures and Tables

**Table 1:** Physicochemical properties of the pharmaceuticals used in this study [45,46].

Pharmaceutical (Formula)	Structure	Mw (g mol^−1^)	Sw (mg L^−1^)	*p*Ka	Log K_ow_	PSA (Å^2^)	HBAC	TS (°C)
Salicylic acid (C_7_H_6_O_3_)	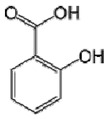	138.12	2.24	(1) 3.0 (2) 13.6	2.2	57.5	3	< 120 [47]
Ibuprofen Sodium (C_3_H_17_NaO_2_)	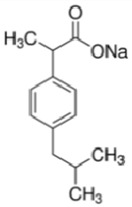	228.26	100.0	4.9	3.8	40.1	2	< 75 [48]
M_W_—molecular weight; Sw—water solubility (25 °C); *p*Ka—acid dissociation constant; (1) *p*Ka_1_; (2) *p*Ka_2_; K_ow_—octanol-water partition coefficient; PSA—polar surface area; HBAC—hydrogen bound acceptor count; TS—thermal stability.								

**Table 2:** Fitted parameters for the kinetic and equilibrium models considered.

		*Scenedesmus Obliquus*		Activated Carbon	
		Salicylic Acid	Ibuprofen	Salicylic Acid	Ibuprofen
**Kinetic equations**					
**Pseudo-first order**	*k_1_* (min^−1^)	0.062 ± 0.006	0.11 ± 0.02	0.22 ± 0.03	0.095 ± 0.007
	*q_e_* (mg g^−1^)	4 ± 1	10.32 ± 0.29	198 ± 4	120 ± 2
	*r^2^*	**0.988**	**0.960**	**0.984**	**0.991**
	*S_y.x_*	2.08	0.738	8.94	8.94
**Pseudo-second order**	*k_2_* (g mg^−1^ min^−1^)	0.0016 ± 0.0001	0.016 ± 0.002	0.0020 ± 0.0002	0.0019 ± 0.0002
	*q_e_* (mg g^−1^)	53.6 ± 0.6	10.9 ± 0.2	211 ± 3	137 ± 3
	*r^2^*	**0.998**	**0.993**	**0.996**	**0.995**
	*S_y.x_*	0.944	0.308	4.62	2.94
**Equilibrium isotherms**					
**Freundlich**	*K_F_* (mg g^−1^ (mg L^−1^) ^−1/n^)	10 ± 2	3.4 ± 0.3	41 ± 8	35 ± 7
	*n*	2.51 ± 0.07	3.7 ± 0.3	2.6 ± 0.4	3.3 ± 0.6
	*r^2^*	**0.945**	**0.992**	**0.931**	**0.923**
	*S_y.x_*	4.57	0.37	20.2	12.4
**Langmuir**	*Q_max_* (mg g^−1^)	63 ± 2	11.9 ± 0.3	250 ± 10	147 ± 6
	*K_L_* (L mg^−1^)	0.070 ± 0.005	0.11 ± 0.01	0.066 ± 0.007	0.09 ± 0.01
	*r^2^*	**0.996**	**0.994**	**0.990**	**0.988**
	*S_y.x_*	1.23	0.32	7.50	4.87

**Table 3:** Thermodynamic parameters determined for the adsorption of salicylic acid and ibuprofen onto *Scenedesmus obliquus* biomass.

	Salicylic Acid			Ibuprofen		
Thermodynamic Parameter	Temperature (^°^C)					
	15	25	35	15	25	35
∆*G*^°^ (kJ mol^−1^)	−22.83	−22.74	−22.18	−24.54	−25.11	−25.33
∆*H*^°^ (kJ mol^−1^)	−32.13			−13.02		
∆*S*^°^ (J mol^−1^ K^−1^)	−32.03			40.18		

**Table 4:** Characteristic parameters of TG/DTG curves determined for *Scenedesmus obliquus* biomass before and after salicylic acid and ibuprofen biosorption.

Step of Decomposition	Parameters ^1^	*S. obliquus*	*S. obliquus* after Biosorption of	
			Salicylic Acid	Ibuprofen
**First**	**T_i_ (°C)**	26.6	28.2	32.5
	**T_TG,max_ (°C)**	64.6	77.4	83.3
	**T_f_ (°C)**	65.2	130.2	105.9
	**DTG_max_ (% mg^−1^)**	−1.2	−1.1	−1.0
**Second**	**T_i_ (°C)**	256.6	246.7	253.3
	**T_TG,max_ (°C)**	307.5	274.1	279.7
	**T_f_ (°C)**	353.0	300.4	307.4
	**DTG_max_ (% mg^−1^)**	−7.7	−8.3	−8.4

T_i_—initial thermal decomposition temperature; T_TG,max_—temperature of maximum rate of mass loss; T_f_—final thermal decomposition temperature detected as mass stabilization; DTG_max_—maximum mass loss rate.

**Table 5:** Proximate analysis of *Scenedesmus obliquus* biomass before and after salicylic acid and ibuprofen biosorption.

Sample	Proximate Analysis (wt. %)			
	Moisture Content	Volatile Matter	Fixed Carbon	Ash Content
***S. obliquus***	6.2	77.9	13.2	2.7
***S. obliquus*** **after salicylic acid biosorption**	6.7	68.9	22.4	2.0
***S. obliquus*** **after ibuprofen biosorption**	7.2	70.8	21.7	0.3

## Supplementary Materials

The following are available online at https://www.mdpi.com/1660-4601/17/10/3707/s1, Figure S1. Species distribution diagram of (a) salicylic acid and (b) ibuprofen as a function of pH (adapted from: [1,2], respectively). Figure S2. Thermogravimetry (TG) together with derivative thermogravimetry (DTG) curves over time of *Scenedesmus obliquus* biomass before biosorption (a); and after salicylic acid (b) or ibuprofen (c) biosorption. Figure S3. Differential scanning calorimetry (DSC) together with derivate differential scanning calorimetry (DDSC) curves of *Scenedesmus obliquus* biomass before biosorption (a); and after salicylic acid (b) or ibuprofen (c) biosorption. Table S1. Physicochemical properties of the commercial activated carbon used as reference (Pulsorb WP260), as provided by the producer (Chemviron Carbon). Table S2. Maximum salicylic acid or ibuprofen biosorption capacities (*Q_max_* (mg g^−1^)) of different materials in the literature. Table S3. Fitted equilibrium parameters on the adsorption of salicylic acid and ibuprofen onto *Scenedesmus* biomass at the different temperatures considered in this work. Table S4. Band assignments of Fourier transform infrared (FT-IR) spectra of *Scenedesmus obliquus* biomass before and after salicylic acid and ibuprofen biosorption. Table S5. Characteristic parameters of differential scanning calorimetry (DSC) together with derivate differential scanning calorimetry (DDSC) curves determined for *Scenedesmus obliquus* biomass before and after salicylic acid and ibuprofen biosorption.
