# Supplementary material for: Green Microalgae Scenedesmus Obliquus Utilization for the Adsorptive Removal of Nonsteroidal Anti-Inflammatory Drugs (NSAIDs) from Water Samples
Source: Int J Environ Res Public Health. 2020 May 25;17(10):3707. doi: 10.3390/ijerph17103707 (PMC7277159; doi:10.3390/ijerph17103707)
Supplement: Supplementary file 1 [file ijerph-17-03707-s001.pdf]

SUPPLEMENTARY INFORMATION

Green Microalgae *Scenedesmus obliquus* Utilization for the Adsorptive Removal of Nonsteroidal Anti-Inflammatory Drugs (NSAIDs) from Water Samples

Andreia Silva [1], Ricardo N. Coimbra [2], Carla Escapa [3], Sónia A. Figueiredo [1], Olga M. Freitas [1] and Marta Otero [2,4,*]

[1] REQUIMTE/LAQV, Instituto Superior de Engenharia Do Porto, Politécnico Do Porto, Rua Dr. António Bernardino de Almeida 431, Porto, 4200-072, Portugal; andreia.silva@graq.isep.ipp.pt (A.S.); saf@isep.ipp.pt (S.A.F.); omf@isep.ipp.pt (O.M.F.)

[2] Department of Environment and Planning, University of Aveiro, Campus Universitário de Santiago, Aveiro, 3810-193, Portugal; ricardo.coimbra@ua.pt (R.N.C.)

[3] Department of Applied Chemistry and Physics, Institute of Environment, Natural Resources and Biodiversity (IMARENABIO), Universidad de León, León, 24071, Spain; carla.escapa@unileon.es (C.E.)

[4] Centre for Environmental and Marine Studies (CESAM), University of Aveiro, Campus Universitário de Santiago, Aveiro, 3810-193, Portugal

* Correspondence: marta.otero@ua.pt; +351 234247094 /ext: 25010 (M.O.)

Contents

Page 2 - Figure S1. Species distribution diagram of (a) salicylic acid and (b) ibuprofen as a function of pH (adapted from: [1] and [2], respectively).

Page 3 - Figure S2. Thermogravimetry (TG) together with derivative thermogravimetry (DTG) curves over time of *Scenedesmus obliquus* biomass before biosorption (a); and after salicylic acid (b) or ibuprofen (c) biosorption.

Page 4 - Figure S3. Differential scanning calorimetry (DSC) together with derivate differential scanning calorimetry (DDSC) curves of *Scenedesmus obliquus* biomass before biosorption (a); and after salicylic acid (b) or ibuprofen (c) biosorption.

Page 5 - Table S1. Physicochemical properties of the commercial activated carbon used as reference (Pulsorb WP260), as provided by the producer (Chemviron Carbon).

Page 6 - Table S2. Maximum salicylic acid or ibuprofen biosorption capacities (Q_{max} (mg g^{-1})) of different materials in the literature.

Page 7 - Table S3. Fitted equilibrium parameters on the adsorption of salicylic acid and ibuprofen onto *Scenedesmus* biomass at the different temperatures considered in this work.

Page 8 - Table S4. Band assignments of Fourier transform infrared (FT-IR) spectra of *Scenedesmus obliquus* biomass before and after salicylic acid and ibuprofen biosorption.

Page 9 - Table S5. Characteristic parameters of differential scanning calorimetry (DSC) together with derivate differential scanning calorimetry (DDSC) curves determined for *Scenedesmus obliquus* biomass before and after salicylic acid and ibuprofen biosorption.

Page 10 - References

International Journal of
Environmental Research and Public Health

Figure S1 depicts the species distribution diagram as a function of pH for salicylic acid and ibuprofen (respectively adapted from [1] and [2]).The pH value impacts the charge of the species in solution [3,4]. In the case of salicylic acid, diprotic species (neutral) are dominant (about 90%) at pH values below 2, monoprotic species (negative) are dominant at pH values between 4 and 13 and completely dissociated species (negative) are dominant (about 70%) at pH values above 13 [2] (see Figure S1(a)) The carboxylic group of ibuprofen is not charged at low solution pH (equal or below to *pK*a). However, as the solution pH increases from 3 to 7, the carboxylic group begins to dissociate and almost all ibuprofen molecules are negatively charged when the solution pH is above 7 [1,5] (see Figure S1(b))

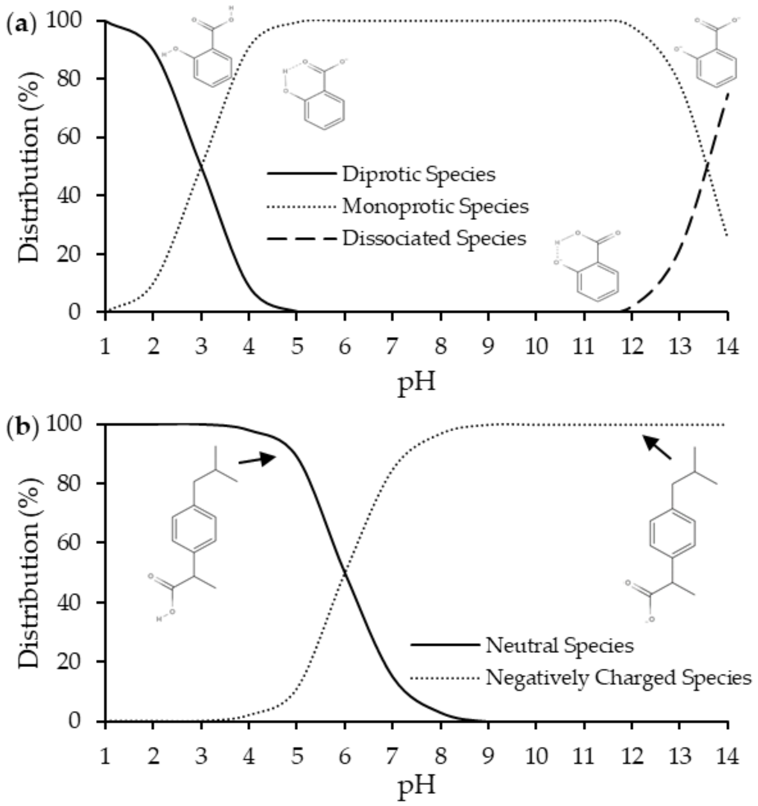

Figure S1. Species distribution diagram of (**a**) salicylic acid and (**b**) ibuprofen as a function of pH (adapted from: [1] and [2], respectively).

International Journal of
Environmental Research and Public Health

MDPI

Simultaneous thermal analysis (STA) allows identifying mass losses and thermal transitions occurred during the decomposition of *Scenedesmus obliquus* biomass before and after pharmaceuticals biosorption through the interpretation of TG/DTG curves over time are shown in Figure S2.

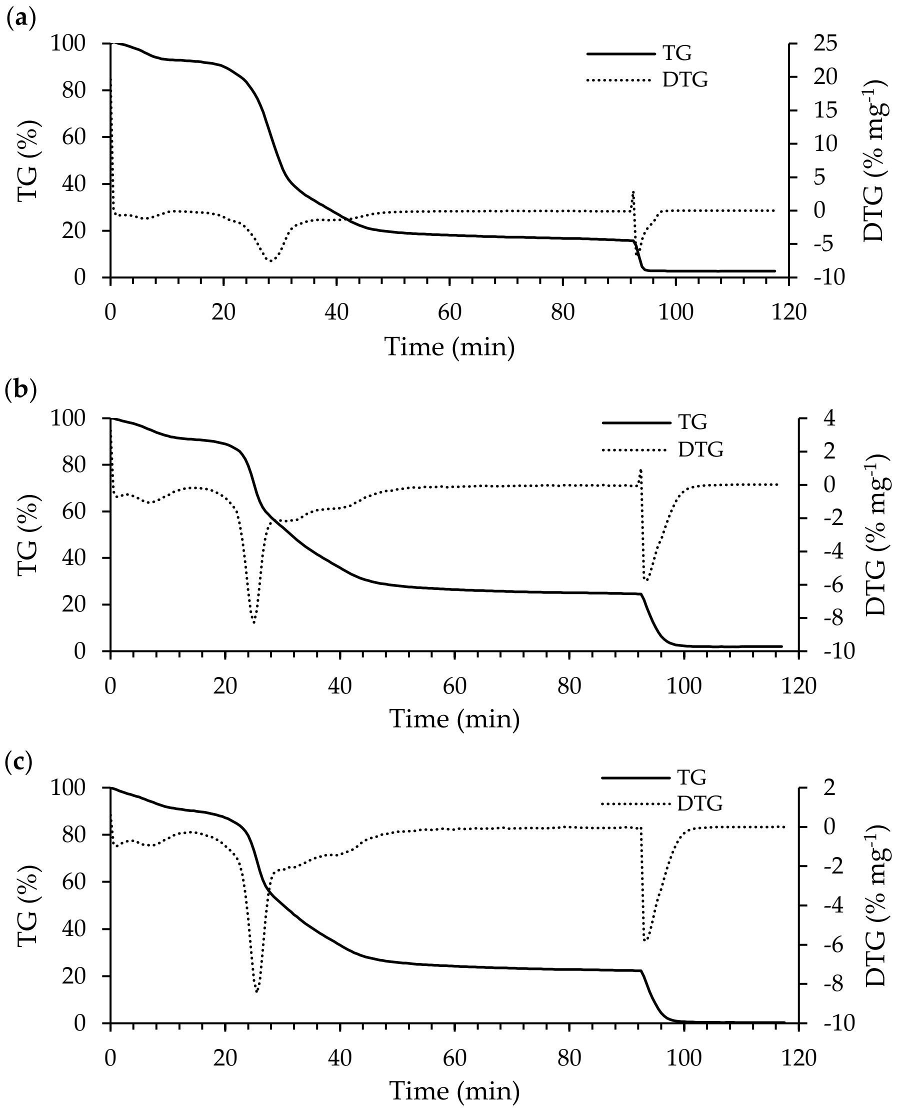

Figure S2. Thermogravimetry (TG) together with derivative thermogravimetry (DTG) curves over time of *Scenedesmus obliquus* biomass before biosorption (**a**); and after salicylic acid (**b**) or ibuprofen (**c**) biosorption.

Differential scanning calorimetry (DSC) together with derivate differential scanning calorimetry (DDSC) curves give complementary information to TG/DTG curves, namely in terms of energy consumption or liberation. Therefore, DSC/DDSC provide more precise information about the occurring transformations on microalgae biomass. The onset temperature (T_{onset}) and the endset temperature (T_{enset}) are, respectively, the temperatures that define the start and the end of a thermal event in DSC/DDSC curves, used to determine the enthalpy (ΔH) by peak integration. The maximum temperature ($T_{DSC,max}$) is the temperature of maximum energy released during a thermal event.

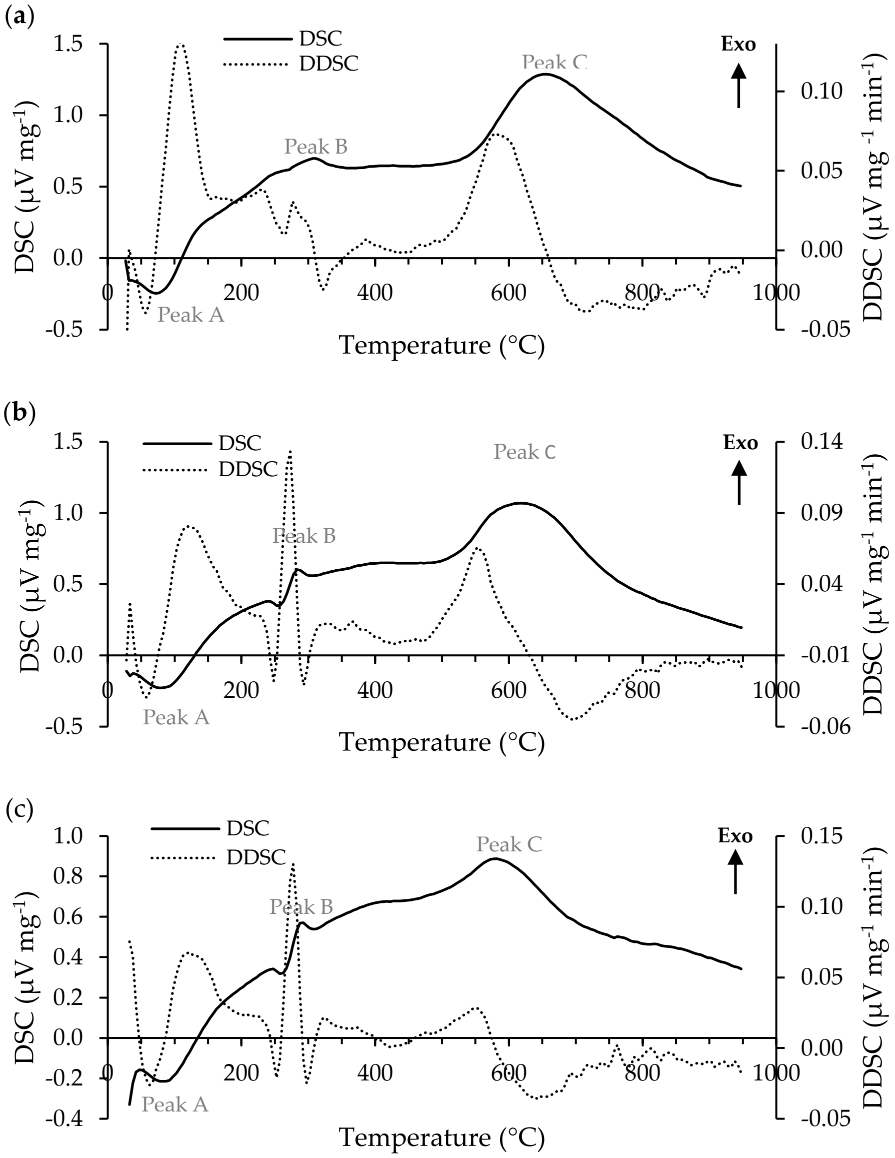

Figure S3. Differential scanning calorimetry (DSC) together with derivate differential scanning calorimetry (DDSC) curves of *Scenedesmus obliquus* biomass before biosorption (**a**); and after salicylic acid or ibuprofen (**b**) biosorption.

International Journal of
Environmental Research and Public Health

Table S1. Physicochemical properties of the commercial activated carbon used as reference (Pulsorb WP260), as provided by the producer (Chemviron Carbon, Feluy, Belgium).

Specifications	
Specific Surface Area, SBET (m^2 g^{-1})	1050
Iodine number (mg g^{-1})	1020
Mean particle diameter (μm)	30
Density, loose packing (kg m^{-3})	250

With respect to other materials used for the biosorption of salicylic acid or ibuprofen from water, Table 2 depicts some recently published Q_{max} and makes evident that the here determined values for *Scenedesmus obliquus* biomass are within the range of values in the literature.

Table S2. Maximum salicylic acid or ibuprofen biosorption capacities (Q_{max} (mg g^{-1})) of different materials in the literature.

Biosorption of Salicylic acid			Biosorption of Ibuprofen		
**Material**	**Q_{max} (mg g^{-1})**	**Reference**	**Material**	**Q_{max} (mg g^{-1})**	**Reference**
Sterile Malmo soil (Alfisols)	18	[6]	Functionalized bean husks	32	[7]
Sterile Drummer soil (Mollisols)	12	[6]	Modified *Scenedesmus obliquus* biomass	42	[8]
Sterile Jefferson (Ultisols)	57	[6]	Knotweed leaves	38	[9]
Scenedesmus obliquus biomass	63	This work	*Phaeodactylum tricornutum* biomass	4	[10]
			Scenedesmus obliquus biomass	12	This work

International Journal of
Environmental Research and Public Health

MDPI

Table S3. Fitted equilibrium parameters on the adsorption of salicylic acid and ibuprofen onto *Scenedesmus* biomass at the different temperatures considered in this work.

		Salicylic acid			Ibuprofen		
		**15°C**	**25°C**	**35°C**	**15°C**	**25°C**	**35°C**
**Freundlich**	K_F (mg g^{-1} (mg L^{-1})$^{-1/n}$)	13 ± 3	10 ± 2	5.8 ± 1.2	4.1 ± 0.6	3.4 ± 0.3	2.7 ± 0.4
	n	2.6 ± 0.4	2.51 ± 0.07	2.0 ± 0.2	3.7 ± 0.5	3.7 ± 0.3	3.8 ± 0.6
	r^2	**0.914**	**0.945**	**0.958**	**0.923**	**0.970**	**0.970**
	$S_{y.x}$	6.57	4.57	3.62	12.4	0.87	0.56
**Langmuir**	Q_{max} (mg g^{-1})	67 ± 3	63 ± 2	62 ± 3	14.1 ± 0.3	11.9 ± 0.3	9.6 ± 0.3
	K_L (L mg^{-1})	0.10 ± 0.02	0.070 ± 0.005	0.066 ± 0.004	0.12 ± 0.01	0.11 ± 0.01	0.087 ± 0.011
	r^2	**0.985**	**0.996**	**0.994**	**0.994**	**0.994**	**0.991**
	$S_{y.x}$	2.76	1.23	1.31	0.37	0.32	0.31

International Journal of
Environmental Research and Public Health
MDPI

Table S4. Band assignments of Fourier transform infrared (FT-IR) spectra of *Scenedesmus obliquus* biomass before and after salicylic acid and ibuprofen biosorption.

Band	Wavenumber (cm^{-1})			Band Assignments [1]	References
	S. obliquus	*S. obliquus* after biosorption of			
		Salicylic acid	Ibuprofen		
A	**3408**	**3405**	3423	*ν*(O-H) (associated) to water molecules or hydroxyl radicals of polysaccharides<br>*ν*(N-H) (associated) to proteins (amide A)	[11–14]<br>[11,12,14]
B	2924	2924	2924	$\nu_{as}(CH_2)$ of lipids	[11–16]
C	2853	2853	-------	$\nu(CH_2)$ of lipids	[11–13,15]
D	1655	1655	1655	*ν*(C=O) of amides I band associated with proteins	[11–15,17]
E	1545	1541	1541	*δ*(N-H) of amides II band and *δ*(C-N) of proteins	[11–17]
F	-------	1458	1458	$\delta_{as}(CH_2)$ of lipids<br>$\delta_{as}(CH_2)$ and $\delta_{as}(CH_3)$ of proteins	[11–14]<br>[11,12,14,15,17]
G	1384	1383	1383	$\delta_s(N(CH_3)_3)$ of lipids<br>$\delta_s(CH_2)$ and $\delta_s(CH_3)$ of proteins, and ν_s(C-O) of carboxylic groups	[11,13,14,16]<br>[11,13–17]
H	1246	1244	1241	ν_{as}(>P=O) of phosphodiester backbone from nucleic acids and phospholipids	[11–15,17]
I	1153	1154	1154	ν_{as}(C-O) of starch and complex sugar ring modes<br>*ν*(C-O-C) of polysaccharides (from carbohydrates)<br>*ν*(Si-O) of silicate frustules	[11,13,14]<br>[13–15,17]<br>[15]
J	1078	1079	1079	ν_{as}(C-O) of starch and complex sugar ring modes<br>ν_{as}(>P=O) of phosphodiester backbone from nucleic acids<br>*ν*(C-O-C) of polysaccharides (from carbohydrates)<br>*ν*(Si-O) of silicate frustules	[1–14,16]<br>[11–14]<br>[13–17]<br>[15,17]
K	1025	1025	1026	ν_{as}(C-O) of starch and complex sugar ring modes<br>ν_{as}(>P=O) of phosphodiester backbone from nucleic acids<br>*ν*(C-O-C) of polysaccharides (from carbohydrates)<br>*ν*(Si-O) of silicate frustules	[11,13,14]<br>[11,13,14]<br>[12–15,17]<br>[15]

[1] *ν* - stretching; ν_s - symmetric stretching; ν_{as} - asymmetric stretching; δ - deformation; δ_s - symmetric deformation; δ_{as} - asymmetric deformation.

Table S5. Characteristic parameters of differential scanning calorimetry (DSC) together with derivate differential scanning calorimetry (DDSC) curves determined for *Scenedesmus obliquus* biomass before and after salicylic acid and ibuprofen biosorption.

Peak	Parameters [1]	*S. obliquus*	*S. obliquus* after biosorption of	
			Salicylic acid	Ibuprofen
A	T_{onset} (°C)	26.9	28.0	72.9
	$T_{DSC,max}$ (°C)	71.9	77.7	84.2
	T_{endset} (°C)	130.2	151.2	146.3
	ΔH (μV mg^{-1})	−131	−101	−17
B	T_{onset} (°C)	223.8	262.8	277.1
	$T_{DSC,max}$ (°C)	309.5	284.4	290.0
	T_{endset} (°C)	343.6	297.9	303.4
	ΔH (μV mg^{-1})	47	17	10
C	T_{onset} (°C)	540.5	517.9	498.1
	$T_{DSC,max}$ (°C)	651.0	618.4	579.4
	T_{endset} (°C)	866.5	758.3	766.4
	ΔH (μV mg^{-1})	3246	1315	592

[1] T_{onset} - onset temperature for energy release and peak integration; $T_{DSC,max}$ - temperature of maximum energy release during process; T_{enset} - endset temperature for energy release during process; ΔH - enthalpy determined by integration of the peak in the corresponding DSC curve.

References

1. Singh, R.; Hankins, N. *Emerging Membrane Technology for Sustainable Water Treatment*, 1st ed.; Elsevier Science: Oxford, UK 2016.
2. Bernal, V.; *et al.* Thermodynamic study of the interactions of salicylic acid and granular activated carbon in solution at different pHs. *Adsorption Science & Technology*, **2017**, 36(3-4), 833-850, doi:10.1177/0263617417730463.
3. Davis, T.A.;*et al.*, A review of the biochemistry of heavy metal biosorption by brown algae. *Water Research*, **2003**, 37(18), 4311-4330, doi:10.1016/S0043-1354(03)00293-8.
4. Ozer, A.;*et al.* Biosorption of Acid Blue 290 (AB 290) and Acid Blue 324 (AB 324) dyes on *Spirogyra rhizopus. J Hazard Mater,* **2006**, 135(1-3), 355-64, doi:10.1016/j.jhazmat.2005.11.080.
5. Oh, S.; *et al.* Effects of pH, dissolved organic matter, and salinity on ibuprofen sorption on sediment. *Environ Sci Pollut Res Int,* **2016**, 23(22), 22882-22889, doi:10.1007/s11356-016-7503-6.
6. Jagadamma, S.; *et al.* Selective sorption of dissolved organic carbon compounds by temperate soils. *PLoS One*, **2012**, 7(11), e50434, doi:10.1371/journal.pone.0050434.
7. Bello, O.S.; *et al.* Biosorption of ibuprofen using functionalized bean husks. S*ustainable Chemistry and Pharmacy*, **2019**, 13, doi:10.1016/j.scp.2019.100151.
8. Ali, M.E.M.; *et al.* Removal of pharmaceutical pollutants from synthetic wastewater using chemically modified biomass of green alga *Scenedesmus obliquus. Ecotoxicol Environ Saf,* **2018**, 151, 144-152, doi:10.1016/j.ecoenv.2018.01.012.
9. Mucha, M.; Mucha, M. Ibuprofen and acetylsalicylic acid biosorption on the leaves of the knotweed Fallopia x bohemica. *New Journal of Chemistry,* **2017**, 41(16), 7953-7959, doi:10.1039/C7NJ01658A
10. Santaeufemia, S.; Torres, E.; Abalde, J. Biosorption of ibuprofen from aqueous solution using living and dead biomass of the microalga *Phaeodactylum tricornutum. Journal of Applied Phycology*, **2017**, 30(1), 471-482, doi:10.1007/s10811-017-1273-5.
11. Sigee, D.C.; *et al.* Fourier-transform infrared spectroscopy of Pediastrum duplex: characterization of a micro-population isolated from a eutrophic lake. *European Journal of Phycology*, **2002**, 37(1), 19-26, doi:10.1017/S0967026201003444.
12. Ponnuswamy, I.; *et al.* Isolation and Characterization of Green Microalgae for Carbon Sequestration, Waste Water Treatment and Bio-fuel Production. *International Journal of Bio-Science and Bio-Technology*, **2013**, 5, 17-26.
13. Grace, C.E.E.; *et al.* Biomolecular transitions and lipid accumulation in green microalgae monitored by FTIR and Raman analysis. *Spectrochim Acta A Mol Biomol Spectrosc*, **2020**, 224, 117382, doi:10.1016/j.saa.2019.117382.
14. Dilek, D. Fourier transform infrared (FTIR) spectroscopy for identification of *Chlorella vulgaris* Beijerinck 1890 and *Scenedesmus obliquus* (Turpin) Kützing 1833. *African Journal of Biotechnology,* **2012**, 11(16), doi:10.5897/AJB11.1863.
15. Giordano, M.; *et al.* Fourier Transform Infrared Spectroscopy as a Novel Tool to Investigate Changes in Intracellular Macromolecular Pools in the Marine Microalga *Chaetoceros Muellerii (Bacillariophyceae). Journal of Phycology,* **2002**, 37(2), 271-279, doi:10.1046/j.1529-8817.2001.037002271.x.
16. Wen, Y.; *et al.* Enantioselective ecotoxicity of the herbicide dichlorprop and complexes formed with chitosan in two fresh water green algae. *J Environ Monit,* **2011**, 13(4), 879-85, doi:10.1039/c0em00593b.
17. Stehfest, K.; *et al.* The application of micro-FTIR spectroscopy to analyze nutrient stress-related changes in biomass composition of phytoplankton algae. *Plant Physiol Biochem*, **2005**, 43(7), 717-26, doi:10.1016/j.plaphy.2005.07.001.

© 2020 by the authors. Submitted for possible open access publication under the terms and conditions of the Creative Commons Attribution (CC BY) license (http://creativecommons.org/licenses/by/4.0/).
